# Diet-related greenhouse gas emissions and major food contributors among Japanese adults: comparison of different calculation methods

**DOI:** 10.1017/S1368980019004750

**Published:** 2021-04

**Authors:** Minami Sugimoto, Kentaro Murakami, Keiko Asakura, Shizuko Masayasu, Satoshi Sasaki

**Affiliations:** 1 Department of Social and Preventive Epidemiology, Division of Health Sciences and Nursing, Graduate School of Medicine, University of Tokyo, Tokyo 113-0033, Japan; 2 Department of Social and Preventive Epidemiology, School of Public Health, University of Tokyo, Tokyo 113-0033, Japan; 3 Department of Environmental and Occupational Health, School of Medicine, Toho University, Tokyo 143-8540, Japan; 4 Ikurien-Naka, Ibaraki 311-0105, Japan

**Keywords:** Greenhouse gas emissions, Life cycle analysis, Input–output table, Self-selected diet, Japanese

## Abstract

**Objective:**

To develop a greenhouse gas emissions (GHGE) database for Japanese foods using three different approaches, compare the results of estimated diet-related GHGE and determine major food contributors among Japanese adults.

**Design:**

Cross-sectional. Three GHGE databases were developed: (1) a literature-based method including a literature review of life cycle assessment studies of Japanese foods and (2) production- and (3) consumption-based input–output tables (IOT)-applied methods using the Japanese IOT. All databases were linked to the Japanese food composition table and food consumption data. Diet-related GHGE was estimated based on each database and the 4-d dietary record data. Diet-related GHGE were compared in both total and food group level between the databases.

**Setting:**

Japan.

**Participants:**

392 healthy adults aged 20–69 years.

**Results:**

The mean diet-related GHGE significantly differed according to the calculation methods: 4145 g CO_2_-equivalent (CO_2_-eq)/d by the literature-based method, 4031 g CO_2_-eq/d by the production-based method and 7392 g CO_2_-eq/d by the consumption-based IOT-applied methods. It significantly differed in food group level as well. Spearman’s correlation coefficients between three methods ranged from 0·82 to 0·86. Irrespective of the methods, the top contributor to GHGE was meat (19·7–28·8 %) followed by fish and seafood (13·8–18·3 %).

**Conclusions:**

Although the identified major food contributors to GHGE were comparable between the three methods, the estimated GHGE values significantly differed by calculation methods. This finding suggested that caution must be taken when interpreting the estimated diet-related GHGE values obtained using the different calculation methods of GHGE.

The food sector contributes about 20–30 % of total greenhouse gas emissions (GHGE)^(1,2)^, which is an important driver of climate change^(3)^. In particular, livestock meat production makes a major contribution to GHGE^(4)^. Given that food production is driven by food consumption demands^(5)^, there is an urgent need to change dietary choices and to make the food system more sustainable^(6–8)^.

A growing body of literature has reported the environmental impact of diet^(9–28)^, using the life cycle GHGE of each food item or food group obtained by life cycle assessment (LCA)^(29)^. There are a variety of approaches to build the databases of food GHGE. Some researchers used the database with bottom-up approach^(20,23,24)^, top-down approach^(9,30–32)^ or hybrid approach^(10–12)^, while others used a literature-based approach, that is, aggregation of the existing individual LCA studies collected with a literature review^(13–18,21)^. The former three approaches could provide standardised data but need much time and resources for their development^(33)^. On the contrary, the latter is a more feasible approach to obtain the data set, especially when there is no appropriate data set using the former approach specified to the target population. However, it has heterogeneity depending on each LCA studies, such as a variety of functional units and system boundaries^(34)^. The fundamental methodological difference of these approaches should be considered when they are applied^(35)^.

Due to difficulty in obtaining actual measurements, previous epidemiological studies have estimated diet-related GHGE by multiplying the GHGE per weight of food from the GHGE database described above with the intake of the food from the dietary data. This suggests that the estimation of diet-related GHGE could depend on the quality of both the GHGE database and dietary data. In terms of dietary data, previous studies have shown that diet-related GHGE could differ depending on the method used to assess dietary intake^(17,36)^. It was also shown that diet-related GHGE could be underestimated due to misreporting of dietary intake, especially underreporting of energy intake (EI)^(22)^, which is a common systematic error in self-reported dietary assessment^(37)^. However, studies examining how the GHGE database affects the result are limited.

There are a few independent previous studies which have estimated diet-related GHGE using similar dietary data and different GHGE databases. Diet-related GHGE among a national representative sample of French adults aged 18–79 years (energy under-reporters excluded) were relatively similar between a report using a literature-based approach by Vieux *et al*.^(15)^ and other reports using a hybrid approach^(11,38)^. The estimation was also similar among participants aged 18–64 years (under-reporters included)^(19)^ using existing GHGE values reviewed by other researchers^(39)^. On the contrary, among the participants of the 2008–2009 UK National Diet and Nutrition Survey, the energy-adjusted diet-related GHGE (kg CO_2_-equivalent (CO_2_-eq)/7560 kJ), determined using the revised GHGE database which included a literature-based approach by Hoolohan *et al*.^(25)^, was higher than the value reported in a previous study conducted by the same research group using a former literature-based database^(40)^. The energy misreporting-adjusted mean value of the 2008–2014 National Diet and Nutrition Survey participants aged ≥19 years^(22)^ determined using another GHGE database including a literature-based approach^(26)^ was between those of the previous two reports, while the non-adjusted mean was 2–3 kg CO_2_-eq/d lower than the above-mentioned values. Similarly, relatively higher mean values were observed in the Dutch National Food Consumption Survey when an improved GHGE database using a bottom-up approach was used^(27)^, compared with previous reports^(20,23,24)^. Moreover, the estimation among a national representative sample of Swedish adults aged 18–80 years (energy misreporters excluded) using the database with a literature-based approach^(28)^ was higher than that among participants aged 18–64 years (misreporters included but supplement consumers excluded)^(19)^ using another GHGE database^(39)^. Thus, to our knowledge, the effect of using different GHGE databases on the GHGE estimation was not fully examined within the same study.

This study aimed to develop a GHGE database for Japanese foods using three different approaches and to compare the results of estimated diet-related GHGE between the calculation methods. The secondary aim was to determine the major food contributor to diet-related GHGE. The result would help to choose the database to use in the future study in considering its limitation and difference with other methods.

## Methods

### Participants

Dietary data from healthy Japanese adults aged 20–69 years living in twenty study areas covering twenty-three of forty-seven prefectures were obtained. The survey was conducted from February to March 2013. The primary objective of this survey was to estimate Na and K excretion from 24-h urine and to identify their food sources^(41,42)^. Details of the study design and participant characteristics have been reported elsewhere^(41,42)^. In short, 791 healthy adults (395 men and 396 women) were recruited from co-workers or co-workers’ family members of research dietitians, who were working in separate welfare facilities and supported the study. The participants were not randomly sampled but were volunteers. The exclusion criteria were (i) licensed dietary or medical provider, (ii) residence in the prefecture or adjacent prefecture in which the facility was located for <6 months, (iii) individuals who were under diet therapy prescribed by a doctor or dietitian at the time of the study or within 1 year before the study, (iv) pregnant or lactating women and (v) individuals with history of educational admission for diabetes mellitus.

### Dietary assessment

To reduce the burden to the participants and the research dietitians who managed and checked the recording sheets, half of the voluntary participants (*n* 400) were asked to complete a diet record over four non-consecutive days. The recording days consisted of three working days and 1 d off. The recording days were not randomly selected but selected with adjustment to each participant’s schedule. Around twenty individuals participated in the diet record survey from each study area with consideration of age and sex; each area included four participants (two men and two women) from each of the five 10-year age bands. Each participant was instructed on how to weigh each food and beverage and asked to record all foods and beverages consumed on the four recording days. If participants ate out and weighing was difficult, they were asked to record the restaurant’s name, name of dishes and whether any food was left uneaten. In total, 392 participants (196 men and 196 women) complied (participation rate 98 %).

All collected records were checked by the research dietitians. The research dietitians assigned food item numbers to all recorded foods and beverages according to the Standard Tables of Food Composition in Japan 2015^(43)^. All records were then rechecked by trained dietitian staff at the survey centre. The mean intake of four assessment days was calculated.

### Assessment of basic characteristics

Body height and weight were measured to the nearest 0·1 cm and 0·1 kg, respectively, by the research dietitians or medical staff in the welfare facilities. Participants wore light clothing and no shoes. BMI was calculated as body weight in kg divided by the square of height in meters (kg/m^2^). Blood pressure was measured by the research dietitians, nurses or participants themselves using sphygmomanometers in the welfare facilities. Physical activity level (PAL) was calculated by dividing metabolic equivalent-hour score by 24 h. Metabolic equivalent-h score was estimated by summing the product of the time spent on each of a range of activities (sleeping, standing, walking, cycling, running and activities causing sweating) with various exercise intensities and metabolic equivalent value for each activity^(44,45)^. Their occupation, educational background and smoking habit were assessed using a questionnaire.

The accuracy of reported EI was evaluated by the ratio of EI:BMR based on the Goldberg cut-off method^(46)^. EI:BMR was calculated by dividing average EI of four-assessment days by BMR calculated using sex-specific equation for Japanese population as follows: BMR = (0·0481 × weight (kg) + 0·0234 × height (cm) – 0·0138 × age – X (men = 0·4235, women = 0·9708)) × 1000/4·186^(47)^. Participants were identified as plausible-, under- and over-reporters of EI according to whether the EI:BMR of individual was within, below or above the 95 % confidence limits for agreement between EI:BMR and the respective PAL. When the PAL for sedentary lifestyle (i.e. 1·55 for men and 1·56 for women) was assumed for all subjects, under-, plausible- and over-reporters were defined as having EI:BMR < 1·02, 1·02–2·35 and >2·35 for men and <1·03, 1·03–2·36 and >2·36 for women, respectively. The reason for this assumption was that there is no objective PAL value other than self-reported PAL described above which was assessed with the questionnaire. Under- or over-reporters were identified in this study but were not excluded from the analysis to avoid bias for that exclusion^(37,48)^.

### Greenhouse gas emissions database for food linking to nutrition composition database

Three methods were used to develop the database of GHGE of Japanese food. One was a literature-based method and two were production- and consumption-based input–output tables (IOT)-applied methods. Details of the methodology for database development of the GHGE of food are described in the online supplementary material. GHGE was expressed as CO_2_-eq.

### Greenhouse gas emissions database based on a literature review

In the literature-based method, the GHGE database was developed using a literature review. The systematic literature search for LCA studies that focused on foods consumed in Japan was conducted in July 2018 across three types of literature: peer-reviewed journal papers, conference proceedings and grey reports. Medline (PubMed), Web of Science and Environmental Science Databases (ProQuest, Ebsco and Google Scholar) were searched for peer-reviewed journal papers and conference proceedings published in English using the key words (‘life cycle assessment’ OR ‘life cycle analys*s’ OR ‘LCA’ OR ‘life cycle’) AND (‘greenhouse gas*’ OR ‘GHG*’ OR ‘carbon dioxide’ OR CO_2_ OR ‘global warming potential’ OR GWP) AND (Japan*) AND ‘plural or single form of food name.’ In total, forty-seven reports were extracted through screening of title and abstract and subsequent full-text check. Because of the lack of LCA data for Japanese food, some values were complemented with a global LCA literature database, that is, the Double Food-Environmental Pyramid model^(49)^. The functional unit was standardised as ‘g CO_2_-eq/g food.’ System boundary was also standardised from farm to regional distribution centres or retail. After standardisation of functional unit and system boundaries, sample means of the GHGE values accounting each food were calculated. As a result, the GHGE database of the literature-based method included 163 foods.

### Greenhouse gas emissions database based on input–output tables in Japan

In the production- and consumption-based IOT-applied methods, GHGE databases were developed using GHGE intensity values and unit price data. In this study, emission intensities were determined using the global link input–output (GLIO) model^(50)^ comprising 804 economic sectors in Japan and 230 foreign countries and regions. The GLIO model describes the relationship between the production and consumption systems of Japan and foreign countries. This model was developed to meet the demand to include the global emissions associated with Japanese commodities, given the high dependence rate of fossil fuels and other resources on foreign countries^(50)^. There were both production- and consumption-based intensity values in the published data expressed as per standard monetary unit (e.g. t CO_2_-eq per million Japanese yen; M JPY) for each sector.

Production-based GHGE for each food item was calculated by multiplying the production costs (i.e. unit prices of products) with producer-based GHGE intensities from the GLIO model. The production value, production volume and unit prices (yen per product weight or volume) of each commodity except for some agricultural products or seafood products were obtained from the ‘Table of Domestic Products by Sector and Commodity (TDP)’ (*Bumonbetsu-Hinmokubetsu Kingaku-hyo* in Japanese)^(51)^. The TDP of the year 2005 (TDP 2005) was used because the GLIO model was calculated based on the 2005 Japanese IOT. When there was no unit price in TDP 2005 for a certain food item, the unit value was calculated using the production value and volume or shipment value and quantity published in National Statistics^(52–57)^.

Consumption-based GHGE for each food item was calculated by multiplying the commodity costs (i.e. unit price of the commodity) with consumer-based GHGE intensities from the GLIO model. The unit price of the commodity was obtained mainly from the 2005 National Retail Price Survey (NRP 2005)^(58)^ according to the year of the IOT used to calculate the GLIO model. This survey is conducted annually in 167 villages, towns and cities, and average prices are calculated as mean values of all survey areas, weighted for population size. For food items that were selected as mainly consumed foods but for which the price data were not available in the NRP 2005, prices were taken from the websites of the nationally distributed supermarkets (Seiyu, AEON and Ito-Yokado, Japan). A food item appearing five times or more in total in the whole record, and total intake of it was more than 100 g was selected as ‘mainly consumed food.’ When more than one food item was selected from the same food subgroup, one food which total intake was largest in the subgroup was further selected. However, the price of vegetables, fruits and seafood could not be obtained from these websites due to seasonality. Thus, the mean price value of the foods in the same food group obtained from NRP 2005 was used.

Consequently, GHGE databases of the IOT-applied method included 354 foods for production-based method and 228 foods for consumption-based method.

### Linkage of the databases and weight adjustment

The GHGE values in each GHGE database were linked to 2231 food items including 2229 food items from the Standard Tables of Food Composition in Japan^(43)^ and two additional food items, ‘water for drinking’ and ‘water for cooking.’ The values for each food were determined systematically, based on extrapolation of comparable products. The steps for data assignment are described in the online supplementary material. All procedure was conducted by one author (M.S.). The GHGE value for ‘tap water’ was assigned to seaweeds, and the GHGE values for major ingredient such as soya beans or tap water were assigned to seasonings, as there were no GHGE values obtained from literature-based method for seaweeds and seasonings. In addition, GHGE value for ‘tap water’ in both IOT methods was assumed zero due to the lack of national representative price value data for tap water. Taking into account the weight change during cooking and from wastage, GHGE values were adjusted based on the wastage rate and weight change rate in the Standard Tables of Food Composition in Japan as needed.

### Statistical analysis

The median of GHGE value for food items (per kg food weight) was calculated by food category as defined by the previous report^(41)^. Median was used because the GHGE values were varied by food items even in the same food group but similar food items in the same food group had the same GHGE values due to data assignment procedure. Foods in the ‘meat’ group were further categorised into beef, pork, chicken, meat products and other meat. Twenty-three food items categorised as ‘ready-made foods’ were assumed to be made at home from scratch and excluded from this calculation by food group but included in the following analysis for diet-related GHGE per person. GHGE values were compared between the calculation methods with Wilcoxon signed-rank test by food groups. *P* values were corrected for multiple comparisons by using the Benjamini–Hochberg approach^(59)^.

With regard to other variables, the mean intake values were calculated from the four consumption days. Based on 2231 food items, diet-related GHGE was calculated by multiplying the GHGE value for food items and mean food intake of four assessment days. It was assumed that all dishes were made at home from scratch. Data were presented as mean (sd) for continuous variables and numbers and percentages of participants for categorical variables. The mean differences in the diet-related GHGE among the three calculation methods (i.e. literature-based method and production- and consumption-based IOT-applied methods) were examined using paired *t* test with both total and food group level. *P* values were corrected for multiple comparisons by using the Benjamini–Hochberg approach^(59)^. Spearman’s correlation coefficients between total diet-related GHGE between calculation methods were also examined. Percentage contribution of each food group to diet-related GHGE was calculated as mean diet-related GHGE from each food group per mean total diet-related GHGE. Percentage contribution from ‘ready-made foods’ (twenty-three food items) was allocated to other food groups according to their ingredients. Statistical analyses were performed using SAS statistical software (version 9.4, SAS Institute). All reported *P* values were two tailed, and corrected *P* value of <0·05 was considered significant.

## Results

Table 1 represents the median and 25th and 75th percentiles of GHGE of foods per food weight. GHGE value of food item significantly differed between the calculation methods in most food groups. Beef had the largest GHGE values in all three databases, but the GHGE value varied by methods. For example, the GHGE value of beef was nearly three times higher in the consumption-based IOT-applied method than in the production-based IOT-applied method.

**Table 1:** Greenhouse gas emissions (GHGE) of each food (kg-CO_2_ eq/kg) in foods included in Standard Tables of Food Composition in Japan

Food group*	Number of foods†				IOT-applied method§					
		Literature-based method‡			Production-based			Consumption-based		
		Median	Percentile		Median	Percentile		Median	Percentile	
			25th	75th		25th	75th		25th	75th
Cereal	170	1·1^a^	0·7	1·9	1·4^b^	0·8	2·8	1·8^c^	1·4	2·4
Potato	53	0·4^a^	0·3	0·4	0·8^b^	0·4	1·2	1·7^c^	1·5	2·0
Sugar	36	0·2^a^	0·2	0·9	2·2^b^	1·0	2·6	1·1^b^	1·1	4·2
Pulse	86	0·7^a^	0·7	1·5	1·3^b^	1·0	1·8	3·6^c^	1·2	7·5
Nuts	45	2·1^a^	2·1	2·4	1·8^b^	1·3	2·1	11·1^c^	6·6	22·1
Vegetables	374	0·7^a^	0·6	0·9	0·9^b^	0·5	1·6	2·4^c^	2·2	3·0
Fruits	146	1·5^a^	0·8	2·6	0·9^b^	0·7	1·3	2·1^c^	1·1	2·9
Mushroom	50	1·3^a^	1·2	1·5	3·3^b^	2·8	4·3	10·8^c^	4·3	17·0
Seaweeds‖	54	0·07^a^	0·07	0·07	6·2^b^	2·3	6·2	21·2^c^	5·4	21·4
Fish and seafood	422	7·9^a^	5·3	14·6	5·7^b^	3·2	11·3	19·1^c^	9·6	29·5
Meat	293	10·9^a^	7·6	45·2	7·2^b^	5·4	25·7	17·7^c^	12·4	72·6
Beef	132	45·2^a^	45·2	45·2	25·7^b^	25·7	25·7	72·6^c^	72·6	72·6
Pork	66	7·6^a^	7·6	7·6	5·4^b^	5·4	5·4	17·7^c^	17·7	17·7
Chicken	48	2·4^a^	2·4	3·4	3·5^b^	3·5	4·9	11·7^c^	11·7	15·4
Other meat	22	16·5^a^	10·9	16·5	4·8^b^	4·6	7·1	44·2^c^	44·2	44·2
Processed meat products	25	10·5^a^	10·5	10·5	7·2^b^	7·2	11·1	12·4^c^	9·4	14·8
Egg	20	1·9^a^	1·9	2·2	1·8^ac^	1·5	3·5	2·1^c^	2·1	2·5
Milk and dairy foods	43	7·4^a^	1·5	8·5	5·4^b^	2·5	7·5	6·4^c^	2·3	16·3
Fat and oils	36	2·2^a^	1·4	2·2	1·7^ab^	1·7	3·0	2·6^c^	2·6	4·9
Confectionary	166	1·4^a^	0·65	2·0	4·7^b^	3·7	5·2	4·4^c^	4·0	6·3
Alcohol beverages	32	2·1^a^	1·3	2·1	1·4^b^	0·6	1·8	2·6^c^	2·3	4·3
Tea and coffee	18	0·4^a^	0·4	0·4	0·6^b^	0·4	10·4	0·5^ab^	0·5	0·9
Sugar-sweetened beverages	30	2·6^a^	1·5	2·8	0·7^b^	0·7	0·8	0·6^c^	0·6	0·6
Seasonings¶	132	0·7^a^	0·2	1·1	1·7^b^	1·0	4·4	4·8^c^	1·2	8·1
Water**	2	0·3^a^	0·07	0·4	0·1^a^	0·0	0·3	0·01^a^	0·00	0·03

CO_2_-eq, carbon dioxide equivalent; IOT, input–output tables.

^a,b,c^Values with unlike superscript letters within a row are significantly different from each other by Wilcoxon’s signed-rank sum tests. *P* values were corrected for multiple comparisons by using the Benjamini–Hochberg approach^(59)^, and statistical significance was determined by a corrected two-sided *P* < 0·05.

* Food group classification was according to Asakura *et al*.^(41)^. Food group ‘ready-made food’ (twenty-three food items) was excluded from this presentation.

† ‘Number of foods’ represents the number of food items in the database which categorised to each food group.

‡ In the ‘literature-based method,’ GHGE database was developed by a literature review of previous research calculating life cycle GHGE of food with life cycle assessment (from cradle to regional distribution centre).

§ In the ‘IOT-applied methods,’ GHGE databases (production-based and consumption-based) were developed based on Japanese input–output table (i.e. the global link input–output model; GLIO model)^(50)^.

‖ In the literature-based method, GHGE values for seaweeds were calculated assuming to the same as those of ‘tap water.’

¶ In the literature-based method, GHGE values for seasonings were calculated using a combined value of ingredients for each food.

** In the IOT-applied methods, the GHGE value for bottled water was obtained but those for tap water was not calculated due to lack of national representative price value data for tap water.

Basic characteristics of the study participants are shown in Table 2. Mean age was 44·5 (sd 13·4) years, and BMI was 23·3 (sd 3·7) kg/m^2^. More than 70 % of participants graduated from vocational school, junior college or university. The number (%) of under- and over-reporters of EI was fourteen (3·6 %; nine men and five women) and nine (2·3 %; three men and six women) of 392 participants, respectively.

**Table 2:** Basic characteristics of 196 Japanese men and 196 women (aged 20–69 years)

	All (*n* 392)		Men (*n* 196)		Women (*n* 196)	
	Mean or *n*	sd or %	Mean or *n*	sd or %	Mean or *n*	sd or %
Age (years)	44·5	13·4	44·7	13·3	44·4	13·5
Body height (cm)	163·9	8·4	170·3	5·4	157·6	5·7
Body weight (kg)	62·9	12·6	69·6	11·3	56·1	10·0
BMI (kg/m^2^)	23·3	3·6	24·0	3·5	22·6	3·7
Systolic blood pressure* (mmHg)	123·5	14·9	127·0	14·1	120·0	14·9
Diastolic blood pressure* (mmHg)	78·0	11·2	80·0	11·8	76·0	10·2
Physical activity level†	1·57	0·23	1·56	0·24	1·58	0·23
Energy intake (kJ/d)	8849	2054	9841	2033	7862	1536
Living area (%)						
Hokkaido and Tohoku	59	15	30	15	29	15
Kanto	79	20	40	20	39	20
Hokuriku and Tokai	37	9	18	9	19	10
Kinki	59	15	29	15	30	15
Chugoku and Shikoku	79	20	39	20	40	20
Kyusyu and Okinawa	79	20	40	20	39	20
Occupation (%)						
Clerical	164	42	91	46	73	37
Nursing care	164	42	77	39	87	44
Medical assistant	12	3	4	2	8	4
Cooking assistant	24	6	6	3	18	9
Others	28	7	18	9	10	5
Educational background (%)						
Junior high school or other	10	3	4	2	6	3
Senior high school	104	27	38	19	66	34
Vocational school or junior college	144	37	56	29	88	45
University or graduate school	134	34	98	50	36	18
Smoking habit (%)						
Non-smoker	220	56	66	34	154	79
Past smoker	71	18	57	29	14	7
Current smoker	101	26	73	37	28	14

* One missing value in men was excluded from the calculation.

† Two missing values in women were excluded from the calculation.

Table 3 represents the total diet-related GHGE as well as the GHGE from each food group. A significant difference was observed in the mean total diet-related GHGE values among the three methods: literature-based (4145 g CO_2_-eq/d), production-based (4145 g CO_2_-eq/d) and consumption-based IOT-applied methods (7392 g CO_2_-eq/d). Diet-related GHGE values significantly differed in almost all food group levels as well between the three methods. The largest difference was observed in total diet-related GHGE and diet-related GHGE from fish and seafood and meat. The top food contributor was meat, followed by fish and seafood, irrespective of the estimation methods. Within the meat group, beef had the largest contribution in all methods. Cereal was the third or fourth largest contributor. Seasonings were a subsequently large contributor in both IOT-applied methods but not in the literature-based method. The Spearman’s correlation coefficients for total diet-related GHGE among the three methods ranged from 0·82 to 0·86.

**Table 3:** Diet-related greenhouse gas emissions (GHGE) (g-CO_2_-eq/d) and contribution of each food group to dietary-related GHGE in 196 Japanese men and 196 women (aged 20–69 years), estimated by literature-based and production- and consumption-based accounting IOT-applied methods

				IOT-applied method†					
	Literature-based method*			Production-based			Consumption-based		
	Mean	sd	%‡	Mean	sd	%‡	Mean	sd	%‡
Total	4145^a^	1425	100	4031^b^	1199	100	7392^c^	2568	100
Food group									
Cereals	499^a^	172	12·0	530^b^	178	13·1	586^c^	213	7·9
Potatoes	35·5^a^	41·9	0·9	176^b^	280	4·4	110^c^	94	1·5
Sugars	12·4^a^	24·7	0·3	33·1^b^	29·7	0·8	43·8^c^	68·8	0·6
Pulses	44·6^a^	52·9	1·1	97·4^b^	84·9	2·4	122^c^	104	1·7
Nuts	6·1^a^	13·8	0·1	6·8^a^	13·6	0·2	34·4^b^	60·2	0·5
Vegetables	195^a^	98·9	4·7	209^b^	114	5·2	476^c^	253	6·4
Fruits and fruits juice	254^a^	321	6·1	98·5^b^	108	2·4	155^c^	178	2·1
Mushrooms	19·4^a^	21·7	0·5	52·8^b^	57·5	1·3	117^c^	129	1·6
Seaweeds§	0·4^a^	0·6	0·0	22·2^b^	40·8	0·5	67·9^c^	117	0·9
Fish and seafood	760^a^	591	18·3	557^b^	565	13·8	1340^c^	1133	18·1
Meat	1162^a^	996	28·0	795^b^	599	19·7	2126^c^	1559	28·8
Beef	696^a^	921	16·8	392^b^	522	9·7	1012^c^	1331	13·7
Pork	258^a^	210	6·2	178^b^	148	4·4	593^c^	484	8·0
Chicken	77·6^a^	74·3	1·9	112^b^	108	2·8	373^c^	358	5·1
Other meat	1·3^a^	15·7	0·0	0·4^a^	4·5	0·0	3·8^a^	42·7	0·1
Processed meat products	130^a^	144	3·1	112^b^	120	2·8	144^c^	155	1·9
Eggs	88·3^a^	49·8	2·1	69·6^b^	39·4	1·7	103·8^c^	60·0	1·4
Milk	212^a^	177	5·1	187^b^	157	4·6	216^b^	175	2·9
Fats and oils	65·3^a^	45·6	1·6	55·1^b^	32·9	1·4	87·1^c^	52·0	1·2
Confectionaries	113^a^	118	2·7	201^b^	180	5·0	219^c^	212	3·0
Alcoholic beverages	153^a^	272	3·7	112^b^	205	2·8	498^c^	1028	6·7
Tea and coffees	219^a^	135	5·3	328^b^	191	8·1	404^c^	248	5·5
Sugar-sweetened beverages	48·6^a^	120	1·2	35·9^b^	74·7	0·9	30·4^c^	72·7	0·4
Seasonings‖	102^a^	62·1	2·5	379^b^	362	9·4	627^c^	630	8·5
Water¶	154^a^	160	3·7	84·5^b^	95·3	2·1	29·2^c^	86·7	0·4

CO_2_-eq, carbon dioxide equivalent; IOT, input–output tables.

^a,b,c^Values with unlike superscript letters within a row are significantly different from each other by Wilcoxon’s signed-rank sum tests. *P* values were corrected for multiple comparisons by using the Benjamini–Hochberg approach^(59)^, and statistical significance was determined by a corrected two-sided *P* < 0·05.

* Literature-based GHGE database was developed by a literature review of previous research calculating life cycle GHGE of food with life cycle assessment (from cradle to regional distribution centre).

† IOT-applied GHGE databases were developed by using Japanese input–output table (i.e. the global link input–output model^(50)^).

‡ Calculated as the mean value of diet-related GHGE with food group level divided by the mean total diet-related GHGE.

§ In the literature-based method, GHGE values for seaweeds were calculated assuming to the same as those of ‘tap water.’

‖ In the literature-based method, GHGE values for seasonings were calculated using a combined value of ingredients for each food.

¶ In the consumption-based IOT-based method, ‘tap water’ was assumed to be ‘0’ because GHGE for tap water could not be calculated for lack of the price value of tap water.

## Discussion

To our knowledge, this is the first study to compare the different methods of calculating the GHGE of food used for estimating the total diet-related GHGE. Although strong correlations were observed among the three methods, the mean total diet-related GHGE significantly differed among the three methods. Moreover, similar results were observed in terms of major contributors, but the means of GHGE in food group level significantly differed depending on the method. These differences between methods resulted from the difference of the GHGE value of each food and the number of emission factors included in each database.

Although it was difficult to compare the results between different studies due to heterogeneity of system boundary and method, mean total diet-related GHGE in the present study obtained using the literature-based method and production-based IOT-applied method was similar to the estimation in the USA^(16)^ and several European countries^(11,15,18–20)^, but slightly higher than the values reported in China and India^(13,14,60)^. Given the limited system boundary in these GHGE databases and the relatively lower extent of energy misreporting of dietary data in this study, total diet-related GHGE with the literature-based method and production-based IOT-applied method could be underestimated. By contrast, the GHGE based on the consumption-based IOT-applied method was much higher than values obtained using the other two methods. This might be because the unit prices of some foods, including fish and seafood and meet, especially beef, for the consumption-based IOT-applied method were much higher than those for the production-based IOT-applied methods, possibly due to the difference in data source. Intensity values from the GLIO model were in the same order, which means that the impact from the post-production stage was relatively small^(50)^. The much higher GHGE values in beef in consumption-based IOT-applied method would be explained with the same reason. Actually, the unit price at consumer derived from the NRP 2005 for beef was higher (3·21–7·70 JPY/g) than that calculated from the Livestock census^(54)^ as production-based unit price (0·679 JPY/g). On the other hand, intensity values of production- and consumption-based were in the same order (e.g. 15·42 *v.* 14·31 t CO_2_eq/M JPY). It is unknown whether the estimation based on consumption-based IOT-applied method is over-estimate or appropriate as there is no available gold standard measurement of diet-related GHGE. It is possible that this estimation is reasonable with the assumption that higher resource consumption in post-production stage results in higher unit price for some food groups. The estimated value needed to be compared with the estimation based on another approach including post-production stage to evaluate the validity in the further study.

Consistent with previous studies from Western countries^(15,19–21,26)^, livestock meat was a major contributor to diet-related GHGE among Japanese people. This suggests strong evidence for the recommendation of reducing the intake of livestock meat and improving the production of livestock meat^(1,2,6–8,61,62)^. On the contrary, cereals and fish and seafood had a large contribution among Japanese adults, while dairy products had a small contribution among this group. This result was similar to that reported in China, where rice and vegetables were major contributors, followed by pork and fish^(14,60)^. In such countries, other strategies should be implemented to achieve a sustainable diet, in addition to replacing animal-based foods with plant-based foods^(63)^. Moreover, diet-related GHGE from seasonings should not be ignored among Japanese. Seasonings were a major source of high Na intake among Japanese people^(41)^. Thus, reducing Na intake from seasonings should be emphasised from both the health and environmental perspectives. In this regard, caution is needed for the data quality of GHGE value for seasonings. The data quality for seasonings was lower than other food items in the literature-based method because the values of ingredients were extrapolated. Besides, the data quality for seasonings in both IOT-applied methods would be similar to other food items because the GHGE values were calculated as with other food items. Based on this, IOT-applied method would be better to assess diet-related GHGE from seasonings.

In many previous studies, diet-related GHGE values were calculated based only on one type of GHGE database^(9,10,19–21,11–18)^. No previous study has compared the diet-related GHGE values obtained using different estimation methods used within the same study. In independent but comparable previous studies, similar or relatively higher diet-related GHGE values were obtained through estimation using the latest database or originally developed database^(11,25,27)^ compared with the estimation using the previous database or those developed by other researchers^(11,19,20,40)^. The reason why relatively higher GHGE values were obtained in these previous studies is unknown. It is also difficult to decide which value was more accurate as there is no gold standard method to estimate diet-related GHGE as mentioned above. However, it is possible that the diet-related GHGE from some foods which was under- or over-estimated with the original database was changed in the later version because of the improvement of calculation or newly calculated GHGE value for some food. In addition, major food contributors were also similar among them. Partly consistent with these previous studies, relatively similar mean values were obtained using the literature-based method and production-based IOT-applied method in this study. Moreover, the three methods had a similar result for identifying major food contributors. However, total diet-related GHGE values were significantly different, and diet-related GHGE values at food group level also differed among the three methods. Thus, the use of different GHGE databases might have a huge effect on the estimation of total diet-related GHGE.

This study had several limitations. First, there are some methodological limitations in the GHGE databases developed in this study. With regard to the literature-based method, heterogeneity was found among aggregated LCA studies, including differences in system boundary, functional unit and simulated model^(34)^. Studies from other countries were also included as only a few studies have been conducted in Japan. In addition, GHGE values for some food categories were not obtained, while IOT-applied methods covered all food categories except for tap water. Moreover, emission from international transportation could not be included in most cases. These would result in underestimation of diet-related GHGE. By contrast, GHGE intensity values from the GLIO model for IOT-applied methods were not represented in the food item level but in the industrial sector level. This means that precision of GHGE value was depending on the quality of the price data, namely food item classification and accuracy of unit price in price data. In particular, in the consumption-based IOT-applied method, food classifications in the NRP 2005 differed from those in the IOT due to different publishers, while that of the TDP cited for the production-based IOT-applied method was based on IOT. Furthermore, there might be misclassification of food items not only in the above method but also in all methods because food classification and descriptions were different for food items in the GHGE data and SFCTJ 2015. Considering these, the production-based IOT-applied method would be more practical and reliable to use in future studies among the Japanese population compared with the other two methods as it covers a wide range of food items and has relatively small classification bias and methodological heterogeneity, although composite dishes were not included.

Second, this study included only the GHGE and did not consider other environmental indicators as well as other sustainable diet factors. A holistic approach employing several indicators^(61)^ as well as a multi-dimensional approach is needed in future studies according to the concept of sustainable diet^(64)^. Various factors regarding environmental, nutritional, economical and socio-cultural, such as land use, water use, biodiversity, diet quality, affordability and cultural continuity, should be included. Assessing sustainable diet level using such various indicators is one of the possible methods^(65)^. However, the GLIO model only included energy use, GHGE, nitrogen oxide and sulphur oxides. In addition, the development of literature-based would be difficult because bottom-up study for food consumed in Japan assessing other indicators was further limited than GHGE. The database should be extended to include other indicators based on other published data set or data sampling. Third, emission from cooking at home, excluding that from cooking rice, was not included in this study due to the differences in cooking methods and equipment used. In addition, the processing stage for ready-to-eat food and eating occasion were not considered though about 45 % dishes were ready-made food, processed food or dishes served at restaurant. Previous studies reported that the cooking stage at home has a 4·8 % (breakfast) to 25 % (lunch) contribution to total CO_2_ emission based on modelled menus in Japan^(66)^, and ready-made foods could have higher emission intensity than home-made meals^(67)^. The exclusion of emission from cooking at home and the assumption that ‘all food was made at home from scratch’ to the ready-made food could cause under-estimation of diet-related GHGE. These processing stages of both at home and outside home should be considered in future studies. Fourth, diet-related GHGE in this study was estimated based on self-reported dietary intake, which was prone to misreporting due to the changes in dietary habits during the assessment period. However, the proportion of under- and over-reporter of EI is relatively small (3·6 and 2·3 %, respectively). Thus, energy misreporting was minimised in this study. On the contrary, there could be some seasonality effect on food choice among participants; diet was assessed only in winter. Previous study reported seasonal difference of nutritional intake among Japanese^(68)^ but monthly difference has not been reported. February and March were often categorised to the same season. Thus, the month when survey was conducted did not consider to be matter for the consumed foods in this study. Finally, the participants were not randomly sampled from the general Japanese population. It was possible that there was some difference in diet among these participants and general Japanese population. However, food intake of our participants was not much different from the general Japanese population. For example, intake of cereal, vegetables, fish and seafood and meat among the participants in this study was 450, 245, 70·2 and 93·3 g/d, respectively, while the range of mean intake in national representative sample of Japanese (20–69 years)^(69)^ was 424–471, 233–304, 56–91 and 77–122 g/d, respectively. In addition, as mentioned in previous studies, basic characteristics of the participants were similar to those of the general participants^(41,42)^.

In conclusion, although similar results were observed in terms of major food contributors to the diet-related GHGE, a significant difference was observed in the estimated mean value of the diet-related GHGE among the three calculation methods using different approaches. This suggests that using the GHGE database developed from different approaches could result in differences in the estimation of the absolute values of diet-related GHGE. Hence, caution is needed when comparing the estimated GHGE values between studies using other GHGE databases.

## Supplementary material

For supplementary material accompanying this paper visit https://doi.org/10.1017/S1368980019004750. click here to view supplementary material
