# Supplementary material for: Diet-related greenhouse gas emissions and major food contributors among Japanese adults: comparison of different calculation methods
Source: Public Health Nutr. 2020 May 11;24(5):973–83. doi: 10.1017/S1368980019004750 (PMC8025089; doi:10.1017/S1368980019004750)
Supplement: Supplementary file 1 [file S1368980019004750sup001.pdf]

Diet-related greenhouse gas emission and major food contributor among Japanese adults: comparison of different calculation methods

Supporting Material

1. Overview ... 2

2. Database development ... 2

   2.1 Literature-based method ... 2

   2.2 Production-based Input-Output Tables-applied method ... 7

   2.3 Consumption-based Input-Output Tables-applied method ... 12

Tables and figures ... 15

References ... 32

1. Overview

This material provides detailed description on the development of the greenhouse gas emission (GHGE) databases for food products consumed in Japan with the following primary purposes: to estimate the diet-related GHGE, to identify the major food contributor among Japanese, and to further investigate the relationship between diet-related GHGE and other variables such as demographic variables, food intake, and diet quality. From this perspective, GHGE values included in the GHGE databases were linked to the food items in Standard Tables of Food Composition in Japan 2015 (STFCJ 2015) [(1)].

GHGE databases were developed based on life cycle assessment (LCA), the reference method in evaluating the environmental impact of products including foods, in three different ways. One was developed using a literature-based method, in which the results of the previous LCA studies on GHGEs of food products consumed in Japan were extracted. Two were developed based on the global link input-output (GLIO) model [(2)], which describes the relationship between the production and consumption systems of Japan and other countries through of international trade. GHGE value of each food was obtained from the literature review or GLIO model, then linked to the food items in the STFCJ 2015 [(1)], which were selected from frequently consumed food among Japanese children and adults. Supplemental Figure 1 summarized the method of database development described below.

2. Database development

2.1 Literature-based method

In the literature-based method, GHGE database was developed by a literature review of existing LCA studies for foods consumed in Japan. When there was no LCA study regarding a particular food from the literature review, LCA data from other countries were also used.

2.1.1 Literature search

In the literature-based method, GHGE database was developed by literature review. The systematic literature search for LCA studies that focused on foods consumed in Japan was completed in July 2018 with the following three types of literature: peer-reviewed journal papers, conference proceedings and grey report. Searches for peer-reviewed journal papers and

conference proceedings in English were completed in MEDLINE (PubMed), Web of Science, Environmental Science Database – ProQuest, Ebsco, and Google Scholar using the keywords ("life cycle assessment" OR "life cycle analys*s" OR "LCA" OR "life cycle") AND ("greenhouse gas*" OR "GHG*" OR "carbon dioxide" OR CO_2 OR "global warming potential" OR GWP) AND (Japan*) AND "*food name (both plural and singular form).*" Searches for literature in Japanese were performed using CiNii and Google scholar. Moreover, two Japanese journals ("*Journal of Life cycle assessment, Japan*" and "*Journal of the Japanese Agricultural Systems Society*") were manually searched. Inclusion criteria and exclusion criteria were as follows.

Inclusion criteria:

- Process LCA studies for the food which is included in the STFCJ 2015 and produced in Japan or imported to Japan.
- LCA studies including at least one "cradle to farm-gate."
- The GHGE value that should be reported in carbon dioxide equivalent (CO_2-eq) or individually for the following three main gases- CO_2, nitrogen oxide (N_2O), and methane (CH_4).
- The study that discloses system boundary, functional unit, and location of production.
- GHGE value that was calculated as Carbon footprint or global warming potential (GWP) in a 100-year horizon (GWP 100)**.**

Exclusion criteria:

- GHGE values that are not presented as CO_2-eq or CO_2-eq value is not able to be estimated because all three main individual gases (CO_2, N_2O and CH_4) are not presented.
- Details about the method of calculation including considered system boundary are not provided.
- Study that is not available in the English or Japanese language.
- Study that presents results for farms or areas as opposed to a functional unit of a food type (e.g. kg of food as consumed).
- LCA values that are reported on feed for livestock.
- GHGE value that was not calculated according to the other indicators (i.e. GWP in a 20-year horizon).

After duplicate articles were removed, title and abstract were screened according to the predefined eligibility criteria. The following data were extracted: author name, study year, publication year, food name, original system boundary, GHGE value, geographic location of the study, LCA approach utilized, farming methods (e.g. conservation or organic) type of literature (e.g. peer-reviewed article, conference report, grey report, etc.), GHGE value or values of the three main greenhouse gases, original functional unit, version of GWP. A total of 47 reports (32 peer-reviewed articles, 9 conference proceeding, 5 reports, 1 University Bulletin; 24 in English and 23 in Japanese) were found (Supplemental Figure 2). The article information and assessed food items are summarized in Supplemental Table 1 and 2, respectively.

2.1.2 Data extraction

GHGE values were extracted from the literature. We used the original values reported in the literature and did not conduct recalculation to the newest GWP reported by using the Intergovernmental Panel on Climate Change Fifth Assessment Report [(3)], because of lack of information on individual emission values for each greenhouse gas. As a result, GHGE values for 38 food items were collected.

2.1.3 Additional search for data complement

GHGE values from other literature or data sources were additionally extracted for some foods because the number of food items with GHGE values obtained from a systematic review were too few to cover the major 310 foods.

As for domestic production food;

Step 1: When the value was reported in Barilla Center for Food & Nutrition (BCFN) data sheet [(4,5)] and its system boundary included production stage, processing stage, and transportation stage, that value was extracted.

Step 2: When there was no value meeting the criteria in Step 1, a further literature search was conducted in Web of Science. In this step, we did not limit the literature with the country or region. If the reported GHGE value was obtained from the literature search, that value was used.

Step 3: When no data were obtained from Step 1 to Step 2, the value was substituted by the value of other foods.

As for imported food:

Step 1: Existing data in BCFN data sheet [(4)] were searched within the country or region which was the primary producer of the targeted food and was ranked in the top three country as importing country of the product to Japan. When the value was reported in BCFN data sheet [(4)], and its system boundary included production stage, processing stage, and transportation stage, that value was extracted.

Step 2: When there was no value meeting the criteria in Step 1, a further literature search was conducted in the Web of Science with the country name of the top three importers. If there was more than one report found, the value reported was extracted.

Step 3: When no report was found in Step 2, we used the value in BCFN data sheet [(4)] without consideration of the country of origin.

Step 4: When there were no data in BCFN data sheet [(4)] meeting Step 3, further literature search was conducted in Web of Science without consideration of the country name.

Step 5: When no data was obtained from step 1 to 4, the value was substituted by the value of other food.

2.1.4 Determination of greenhouse gas emission (GHGE) value for each food

The functional unit of the original GHGE value extracted from the articles was standardized as “g CO_2-eq/g food.” When the original functional unit was represented as “per farm area” and the yield per area was also described in the article, per mass GHGE value was calculated. System boundary was also standardized to “from farm to the regional distribution center or retail.” When the original literature included only the production stage as system boundary, GHGE from the post-farm stage was added to the original value according to the previous literature (see Supplemental Table 3). Transport from retail to consumer’s home, cooking at home, and the management of food waste and the waste of package were excluded from the system boundary due to the lack of data regarding these processes corresponding to dietary data and LCA study. Furthermore, between-person variation of distance from the retail to the consumer’s home, means of transportation, cooking method and equipment made it difficult to

take these processes into account. However, the cooking method of rice was almost the same in any household, and the emission from the rice cooking was considered according to the existing LCA data [(6)].

After the functional unit and system boundaries were standardized, the sample means of the GHGE value accounting each food were calculated. Different cultivation or feeding method (i.e. conservation and organic) were treated in the same manner. As a result, GHGE values for 163 foods were obtained. These 163 GHGE values were assigned to 2231 food items including 2229 food items in the Standard Tables of Food Composition in Japan 2015 (STFCJ 2015) [(1)] and additional two food items "water for cooking" and "water for drinking," which is not originally included in STFCJ. The values were determined according to the following five-step method.

Step 1: When GHGE values were available from the literatures, that value was assigned to the same food or different form of the same food in the STFCJ 2015 (n=910). For example, GHGE value for "white potato" was assigned to both "white potato, raw" (STFCJ 2015 food code: 2017) and "white potato, boiled" (STFCJ 2015 food code: 2019). GHGE values from literature were also assigned to "water for cooking" and "water for drinking."

Step 2: When GHGE value was not available in step 1 but the GHGE values for the food which was made with a similar production method to the food in STFCJ 2015 or for the food being the major ingredients of the food in STFCJ 2015 were available from the literatures, that available values were assigned (n=637). For example, GHGE value for "tomato" was assigned to "tomato, canned, without salt" (STFCJ 2015 food code: 6184).

Step 3: When GHGE value was not available in step 2 for the food items in STFCJ 2015 composed of single ingredients, the mean value of the GHGE of the same food group was assigned (n=508). For example, the mean value of GHGE values for vegetables was assigned to "celery" (STFCJ 2015 food code: 6119).

Step 4: When GHGE value was not available in step 2 for the food items in STFCJ 2015 composed of several ingredients (e.g. "carry, retort," STFCJ 2015 food code: 18001), the value was calculated according to the recipe data provided in STFCJ 2015 and nutrition composition data of products. (n=69)

Step 5: When GHGE value was not available in the above step, GHGE value for tap water was assigned. Especially, there was no GHGE value for seaweed from the literature, the GHGE

value for seaweed and seaweed products were assigned with the value for “tap water.” (n=106)

Step 6: GHGE values for “*breast milk*” was assumes as “0.” (n=1)

2.1.5 Weight basis adjustments

To take into account the weight change during cooking and wastage, GHGE values were adjusted by the wastage rate and weight change rate with STFCJ 2015 as needed.

2.2 Production-based Input-Output Tables-applied method

In the Input-Output Table (IOTs)-applied method, GHGE database was developed based on emission intensity value from the production-based GLIO model [2,7] and price data of each food item.

2.2.1 Data sources

Production-based greenhouse gas emission ($GHGE_P$) for each food item was calculated by multiplying the production costs by GHGE intensities based on the producer price. In this study, GHGE intensities were determined using the GLIO model [2,7]. GHGE intensities were expressed as per standard monetary unit (e.g. t CO_2-eq per million Japanese yen; [M-JPY]) for each sector. The production value, production volume, and unit prices (yen per product weight [PW] or volume) of each commodity except for some agricultural products or seafood products included in the sectors could be obtained from the “Table of Domestic Products by Sector and Commodity (TDP)” (“*Bumonbetsu-Hinmokubetsu Kingaku-hyo*” in Japanese, Ministry of Internal Affairs and Communications) attached to the Japanese input-output tables. TDP of the year 2005 (TDP 2005) was used because GLIO model was calculated based on Japanese IOTs for 2005.

$GHGE_P$ ($C_{k,i}$) [t CO_2-eq/PW] = $GHGE_P$ (S_k) [t CO_2-eq/M-JPY] * UP_P ($C_{k,i}$) [M-JPY/PW]

where, $GHGE_P$ (X) is the producer price-based GHGE per PW for X, S_k is the sector k, $C_{k,i}$ is the food commodity i included in S_k, and UP_P (X) is the unit price for X. This equation assumed that the environmental burden generated from the commodity is in proportion to its price, i.e., the production cost. [2,7].

2.2.2 Data complement

TDP represents sector name, sub-sector name, production volume, production value, and unit price for each commodity. However, production volume and unit price were not described in TDP 2005 for several agricultural products, seafood products, and alcoholic beverages. When the unit price for $C_{k,i}$ was described in TDP 2005, that unit price was used to calculate GHGE value for product *i*. When the commodity name for a product in TDP 2005 was available, with neither its production volume nor its unit price, the unit price was complemented by using the National Statistics. In this case, production volume for each commodity was retrieved from the National Statistics. Then, unit price was calculated as the quotient of production value per production volume. The National Statistics used for this complement are shown in Supplemental Table 4. The production volume on the National Statistics was cited only when the products in the National statistics were identified as similar with the product described in the TDP and the production volume in that National Statistics for 2000 was same as the production volume in TDP for the year 2000 (TDP 2000). Note that the number of missing data for unit price and production volume was smaller in TDP 2000 than in TDP 2005. Thus, production volume in TDP 2000 was used to check the validity of using the National Statistics for data complement. For salt and alcoholic beverages, we could not find any National Statistics describing the production volumes consistent with the production volume listed in TDP 2000. Therefore, the shipment unit price (M-JPY/t) calculated as the quotient of the shipment value per shipment quantity which was both obtained from the Census of Manufactures ("Kogyo-Toukei," Ministry of Internal Affairs and Communications) was used as a substitute value. The shipment quantity in the Census of Manufactures and the production volume of the TDP were different but had the same order figures. As for some vegetables and fruits (strawberry, watermelon, melon, cucumber, tomato, eggplant, pumpkin, lettuce, and bell pepper), production values were described using cultivation method (open ground and house) in TDP. On the contrary, the Crop Survey (Ministry of Agriculture, Forestry and Fisheries) described the production volume of these vegetables and fruits in total volume (i.e., production volume by open ground plus production by volume house). Thus, production values in TDP were summed by the type of vegetables or fruits (production value by open

ground plus production by value house) and divided by total production volume to obtain the unit price.

The values of the production volume were standardized to “per production weight.” For several commodities expressing the values as “per volume” OR “per slice,” “per production weight” was calculated using density values or reference amount reported in the STFCJ 2015 or FAO/INFOODS Density Database [(8)]. If the density value was not available for the food from the previous report, density was calculated assuming 1 ml = 1 g. After data complement, the GHGE value for each TDP food commodity (t CO_2-eq/t) was obtained by multiplying the unit price for each commodity (M-JPY/t) by the embodied GHG value for each sector from the GLIO model (t CO_2-eq/million yen) including the commodity.

There were no production volume and unit price in TDP for imported food because TDP was the statistical table for the domestic production. Mainly imported foods, such as banana and avocado, were not described in TDP, even their commodity name. To take into these imported foods, we calculated the unit price by dividing imported price by imported volume obtained from the National Trade Census 2005 (Ministry of Finance). The process of this data complement was partly described in Supplemental Figure 3.

2.2.3 Determination of GHGE value for each food

Production-based GHGE for each food item was obtained by multiplying production-based intensity value by sector in GLIO model and food price data of each food items. Consequently, GHGE values for 354 food items were obtained. GHGE values for 2231 food items including 2229 food items in the STFCJ 2015 [(1)] and additional two food items “water for cooking” and “water for drinking,” which is not originally included in STFCJ 2015. The values were determined according to the following eight-step method.

Step 1: When only one identical food commodity existed in TDP for the STFCJ 2015 food items, that TDP food commodity assigned. Food commodity “mineral water” in TDP was assigned to “water for drinking.” (in total, n=1564)

Step 2: When more than two commodities existed in TDP for one food item in STFCJ 2015, and these TDP commodities were included in the same sectors in TDP, embodied values were calculated and were assigned as follows:

$GHGE_P(F_m)$ [t CO_2-eq/t] = $GHGE_P(S_k)$ [t CO_2-eq/M-JPY] * $\sum_{i=1}^{i=n} PVL(C_{k,i}) / \sum_{i=1}^{i=n} PWT(C_{k,i})$ [M-JPY/t]

where $GHGE_P(X)$ is the producer based GHGE for X, F_m is the food *m*, S_k is the sector *k*, $C_{k,i}$ is the product value of commodity $C_{k,i}$, *n* is number of TDP commodities identified, and PVL(X) and PWT(X) are the product value and volume for product X. For example, for "tomato, raw" (STFCJ 2015 code = 6182), three commodity items included in the sector *"Fruits"*, "tomato, open-field culture" (TDP commodity code = 113010107), and "tomato, greenhouse culture" (TDP commodity code = 113020107) were identified. In this case, embodied unit price was calculated as the summed production value divided by the summed production volume of three commodities. Then, GHGE value for "tomato, raw" was calculated as GHGE value for sector *"Fruits"* multiplied by the embodied unit price. (n=51)

Step3: When more than two food commodities in TDP were identified and these commodities were included in different sectors, their mean values were selected as the GHGE values. For example, two commodities "salmon/ *Sea surface fishery* sector" (TDP commodity code= 1710111051) and "salmon/ *Inner water surface fishery* sector" (TDP commodity code= 312010001) were identified for "chum salmon, raw" (STFCJ 2015 code= 10132). Thus, mean GHGE value of "salmon/ *Sea surface fishery* sector" and "salmon/ *Inner water surface fishery* sector" was used as the GHGE value for "chum salmon, raw." As for tea and coffee, two commodities in different forms were identified. In this case, GHGE value was adjusted to the weight of the beverage form, then their mean value were selected. For example, "green tea, leaves/ *Tea and coffee* sector" (TDP commodity code=1129011101) and "green tea beverage/ *Beverage* sector" (TDP commodity code=1129021301) were identified as "green tea, sencha, infusion" (STFCJ 2015 code=16036). Assuming that tea infusion was made by 10g tea and 430 ml hot water according to the STFCJ 2015, GHGE value of "green tea, infusion" was obtained by multiplying the GHGE value for "green tea, leaves" by 10/440. Then, GHGE value for "green tea, sencha, infusion" was calculated as the mean value of "green tea, infusion" and "green tea beverage" (n=41).

Step 4: When identical food commodity was not available in TDP but was available in the National Trade Census 2005, the GHGE value calculated by multiplying embodied GHGE

value from GLIO model by unit price obtained from the imported value and the imported value was assigned. (n=4)

Step 5: When no identical food commodity was available in TDP, a similar commodity with comparable producing or processing process was assigned. (n=316)

Step 6: When there was no similar item available in TDP, and the food was composed of single ingredient, embodied GHGE value obtained from the commodities included in the same sector was assigned (n=230).

Step 7: When there was no similar item available in TDP, and the food was composed of the multiple ingredients, the values were calculated from the recipe from Food Commodity Intake Database because there was no standard recipe database in Japan. (n=23)

Step 8: GHGE values for "water" was assumed "0" due to lack of appropriate data for tap water to calculate unit price. GHGE values for "*breast milk*" was also assumes as "0." (n=2)

Japanese self-sufficiency was low except for rice, vegetables, potatoes, some fruits, milk and egg, and many foods were imported from abroad. Unfortunately, there were no detailed data on the self-sufficiency of each food. Additionally, the food classification of TDP and National Trade Census was inconsistent. Therefore, we could not take into account all imported foods. Unit price from TDP was assigned to calculate GHGE value for STFCJ 2015 food items, regardless of the proportion of the food that was imported from abroad except for a few cases described in Step 4. For example, unit price from TDP was used to calculate the GHGE value for soybeans whose self-sufficiency rate was 51% assuming that the unit price of the imported soybeans was similar to that of the domestic soybeans.

2.2.4 Weight basis adjustments

The crude GHGE value obtained by multiplying the embodied GHGE from GLIO for each sector by the cost of each commodity was based on the uncooked products including inedible parts such as vegetable skin, seed, and fish born. To consider the weight change during cooking and from wastage, GHGE values were adjusted by the wastage rate and weight change rate with STFCJ 2015.

2.3 Consumption-based Input-Output Tables-applied method

IOTs-applied method, GHGE database was developed based on emission intensity value from the consumption-based GLIO model [2,7] and price data of each food item.

2.3.1 Data sources

Consumption-based greenhouse gas emission ($GHGE_C$) for each food item was calculated by multiplying the costs by GHG emission intensities based on the purchase price for household consumption expenditure. GHG emission intensities were also determined using GLIO model [7].

$GHGE_C(C_{k,i})$ [t CO_2-eq/PW] = $GHGE_C(S_k)$ [t CO_2-eq/M-JPY] * $UPc(C_{k,i})$ [M-JPY/PW]

where, $GHGE_C$ (X) is the consumption-based GHGE per PW for X, S_k is the sector k, $C_{k,i}$ is the food commodity i included in S_k, and UP_C (X) is the unit price for X. This equation assumed that environmental burden generated from the commodity is in proportion to its price, i.e., the production cost. UP_C was obtained mainly from the National Retail Price Survey for the year 2005 (NRP 2005) (Statistics Bureau, Ministry of Internal Affairs and Communication) according to the year of the IOTs used to calculate GLIO model. This survey is conducted annually in 167 villages, towns and cities, and average prices were calculated as mean values of all survey areas, weighted for population size in 2004. Population size data of 2004 were used instead of the 2005 data because some villages, towns and cities were merged with the neighboring municipality during 2004-2005 and population size before annexation was not described in the census in 2005. To calculate $GHGE_C$, food commodity in NRP 2005 was assigned to the sector according to the commodity classification in TDP. When the multiple sectors were identified, $GHGE_C$ was calculated by multiplying UP_c obtained from the NRP 2005 by the mean of the embodied GHGE values for identified sectors. When the product was not expressed per weight, it was adjusted to per weight value by using the portion size provided by the NRP 2005 or density provided by the STFCJ 2015. When the density was not available from the STFCJ 2015, the density value from FAO/INFOODS Density Database [8] was used.

2.3.2 Data complements

For food items selected as the mainly consumed food but whose price was not identified in the NRP 2005, prices were taken from the websites of the nationally distributed supermarket (Seiyu, AEON, and Ito-Yokado Japan). However, the price of vegetables, fruits, and seafood could not be obtained from the website because of the seasonality. As a result, food prices of 12 food item were obtained from the websites.

2.3.3 Determination of GHGE value for each food

Consumption-based GHGE for each food items was obtained by multiplying consumption-based intensity value by sector in GLIO model and food price data of each food items. Consequently, GHGE values for 228 food items were obtained. The GHGE values for 2231 food items including 2229 food items in the Standard Tables of Food Composition in Japan 2015 (STFCJ 2015) (1) and additional two food items "water for cooking" and "water for drinking," which is not originally included in STFCJ were determined according to the following three-step method. The values were determined by assigning the food commodities in NRP 2005 and additional food price data were obtained above.

Step 1: When there was only one identical food commodity in the NRP 2005 or the complemented price data with the website, that UPc was assigned. (n=1009).

Step 2: When no identical food was available in the NRP 2005 or the complemented price data, UPc of the similar commodity with comparable producing or processing process was assigned (n=650).

Step 3: When there was no similar item available in the NRP 2005 or the complemented price data, mean value of UPc of the same food group was assigned (n=547).

Step 4: For foods composed of several ingredients, the value was calculated according to the recipe data provided in STFCJ 2015 and nutrition composition data of the products (n=23).

Step 5: GHGE values for "water for coking" was assumed "0" due to lack of appropriate data for tap water to calculate unit price. GHGE values for "*breast milk*" was also assumes as "0" (n=2).

2.3.4 Weight basis adjustments

The crude GHGE value obtained by multiplying the embodied GHGE from GLIO for each sector by the cost of each commodity was based on the products at the discount sale. To consider the weight change during cooking and from wastage, GHGE values were adjusted by the wastage rate and weight change rate with STFCJ 2015as needed.

Tables and figures

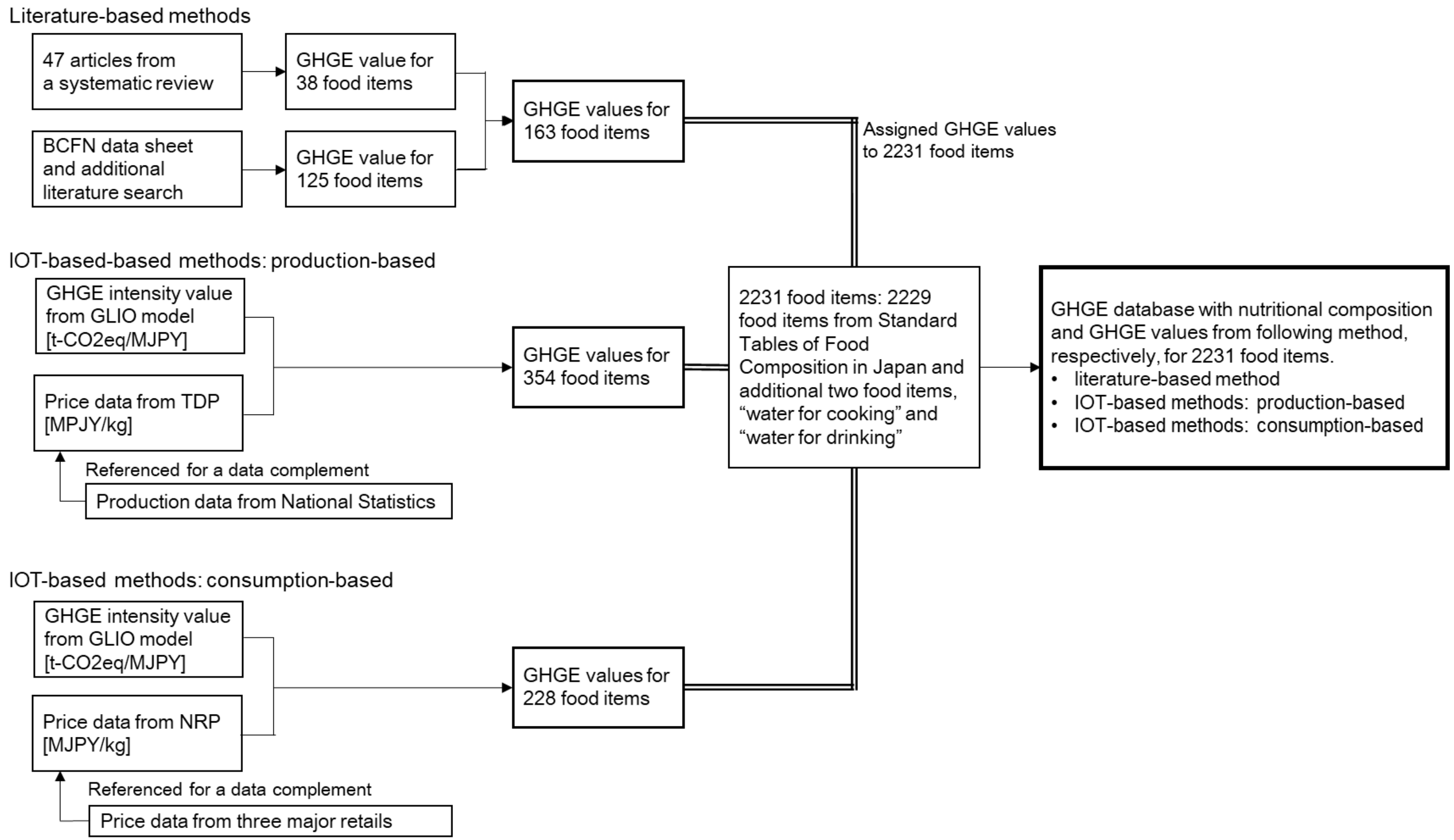

Supplemental Figure 1. Summary of method to develop the greenhouse gas emission databases

IOTs, Input-Output Tables; GHGE, greenhouse gas emissions; GLIO, the global link input-output model [(2)]; TDP, Table of Domestic Products by Sector and Commodity (*Bumonbetsu-Hinmokubetsu Kingaku-hyo,* Ministry of Internal Affairs and Communications); BCFN, Barilla Center for Food & Nutrition; MPJY, million Japanese yen; NRP, the National Retail Price Survey.

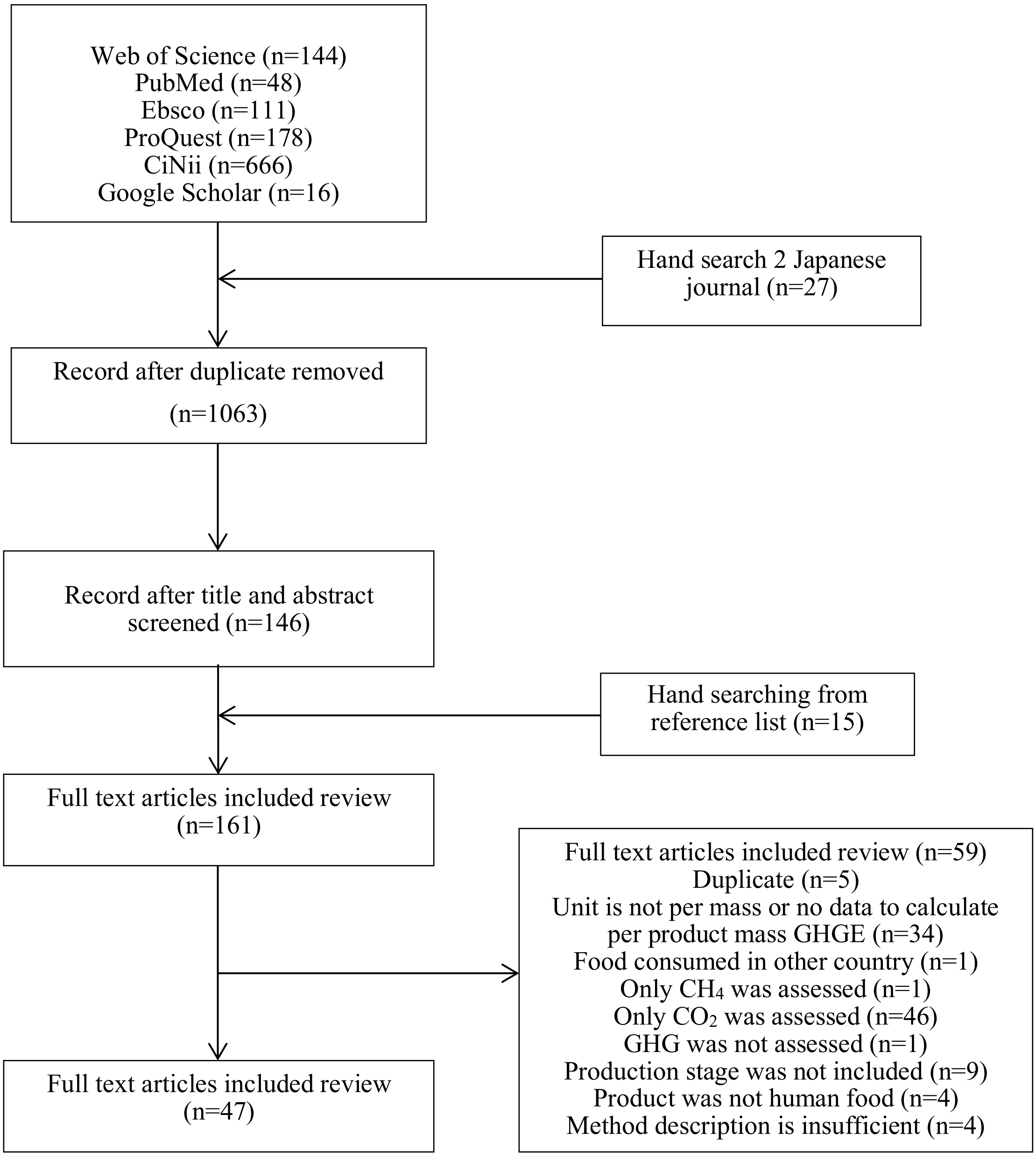

Supplemental Figure 2. Flow diagram for eligible study selection of a systematic review for development of literature-based greenhouse gas emission database.

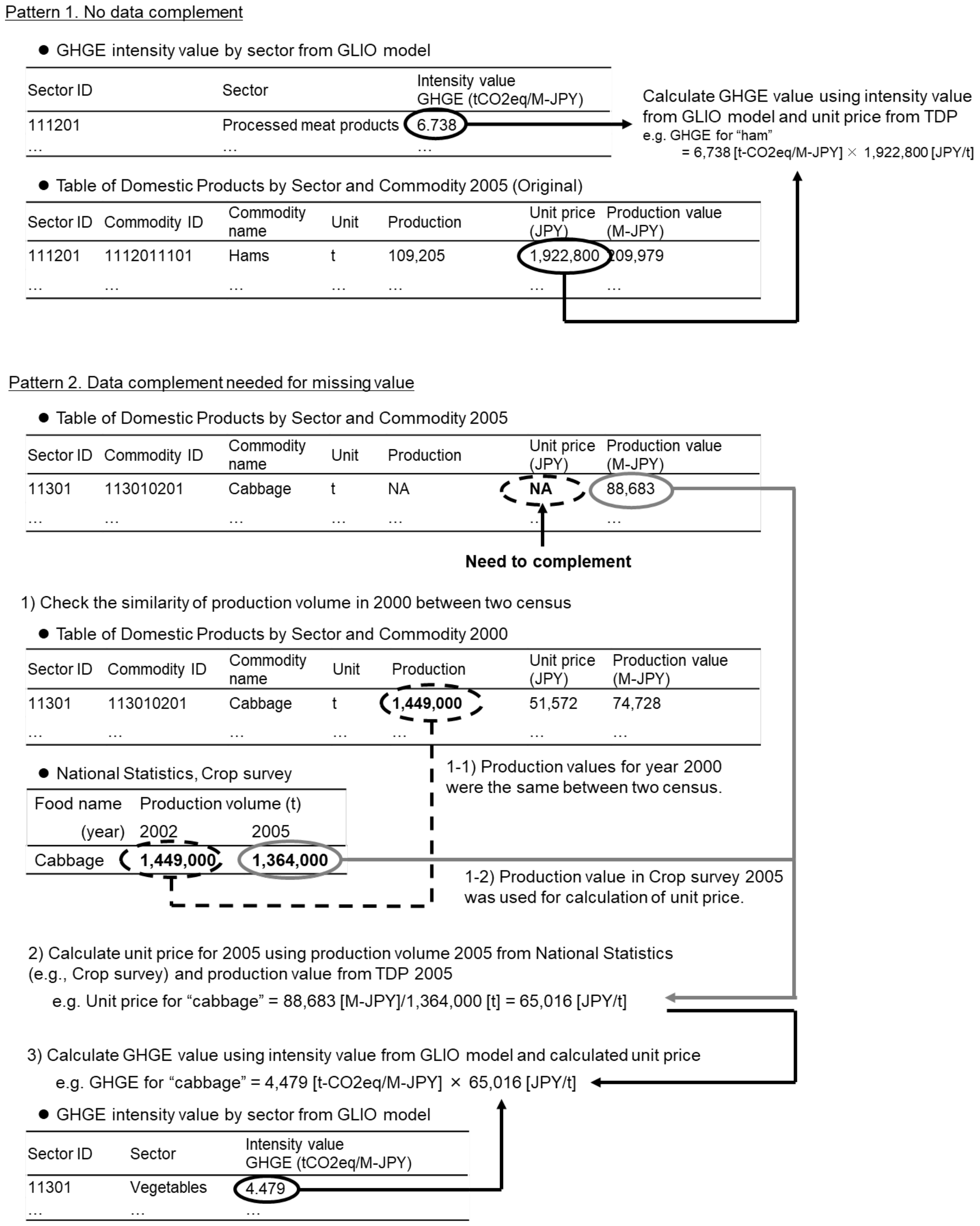

Supplemental Figure 3. The method of data complement in production-based Input-Output Table-applied method.

GHGE, greenhouse gas emissions; JPY, Japanese yen; M-JPY, million JPY; GLIO, the global link input-output model [(2)]; TDP, Table of Domestic Products by Sector and Commodity (*Bumonbetsu-Hinmokubetsu Kingaku-hyo,* Ministry of Internal Affairs and Communications). Note; Pattern 1, when unit value existed in TDP 2005, GHGE for the food item was calculated using

the unit value from TDP 2005 and intensity value from GLIO model. Pattern2, when unit value and production value did not exist in TDP 2005, the unit value was calculated using the other National Statistics with three steps. First, search the National Statistics describing the production data for the targeted food. Then, the production volume for 2000 in TDP 2000 and other National Statistics (e.g., Crop survey) were compared to check the validity of using the National Statistics. If these production volumes were the same value, the production volume in 2005 were extracted from that National Statistics. Next, unit price for 2005 was calculated by dividing production value from TDP by production volume from the National Statistics. Lastly, GHGE for the food item was calculated using the calculated unit value and intensity value from GLIO model.

Supplemental Table 1 Summary of selected articles

Reference	Article type	Language	Food assessed	System boundary	Sources of emissions
Ando T, Yoshikawa N. Estimation of the carbon footprint of conventional rice (variety: Koshihikari produced in Toso area, Chiba Prefecture). J Life Cycle Assessment, Japan. 2011;7:387–95.	Journal article	Japanese	White rice	Cradle to grave	Seeds production, fertilizer and pesticide production, emission from paddy field, fuel, electricity, packaging material, transportation, cooking, waste of package
Beccali M, Cellura M, Iudicello M, Mistretta M. Life cycle assessment of Italian citrus-based products. Sensitivity analysis and improvement scenarios. J Environ Manage. 2010;91:1415–28.	Journal article	English	Citrus juice	Cradle to gate	Production and use of fuel, fertilizers, herbicides and pesticides, fuel, electricity, water, cooling water
Donnell BO, Goodchild A, Cooper J, Ozawa T. The relative contribution of transportation to supply chain greenhouse gas emissions: A case study of American wheat. Transp Res Part D. 2009;14:487–92.	Journal article	English	Wheat	Cradle to point of use	Energy production, production and use of fertilizer, herbicide and insecticide during wheat cultivation, wheat transportation from the farm to Japan
Hassard HA, Couch MH, Techa-erawan T, McLellan BC. Product carbon footprint and energy analysis of alternative coffee products in Japan. J Clean Prod. 2014;73:310–21.	Journal article	English	Coffee	Cradle to grave	Production and use of fertilizer, emissions during coffee cultivation, shipping containers, the energy use in the roasting process electricity, package
Hayashi K. Ecological-economic assessment of farms using multi-input multi-output models: life cycle assessment with multiple paired comparisons. Int J Sustain Dev. 2014;17:9.	Journal article	English	Rice	Cradle to gate	Production and use of fertilizer and pesticide, emission from paddy field, farm operation

Reference	Article type	Language	Food assessed	System boundary	Sources of emissions
Hirai Y, Suenaga T, Hahagami K. Energy consumption and greenhouse gas emissions associated with rice production in rice terraces. J Japanese Agric Syst Soc. 2012;28:47–56.	Journal article	Japanese	Brown rice	Cradle to gate	Production and use of fertilizer and pesticide, emission from paddy field, fuel, electricity, agricultural machinery
Hishinuma T, Kurishima H, Genchi Y. An LCA of greenhouse gas emissions at pork production utilizing food residues as feed. 5th Meet Inst Life Cycle Assessment, Japan. 2010;12–3.	Conference Proceeding	Japanese	Pork	Cradle to retail	Feed production, animal management, manure treatment、slaughter、electricity, fuel, disinfectant use
Hishinuma T, Kurishima H, Yutaka G. An LCA of greenhouse gas emissions at pork production utilizing food residues as feed. The 6th meeting of the institute of Life Cycle Assessment, Japan. 2011. p. 394–5.	Conference Proceeding	Japanese	Pork	Cradle to retail	Feed production, animal management, manure treatment、slaughter、electricity, fuel, disinfectant use
Hishinuma T. Life Cycle Assessment of Greenhouse Gas Emissions from a Pork production System in Japan. Environmental Information Science. 2015. 29. 159-164	Journal article	Japanese	Pork	Cradle to retail	Feed production, animal management, manure treatment、slaughter、electricity, fuel, disinfectant use、enteric fermentation, composting treatment
Hokazono S, Hayashi K. Comparative life cycle assessment of organic and conventional soybean production in paddy fields under rotational cropping. J Life Cycle Assessment, Japan. 2012;8:2–13.	Journal article	Japanese	Soybean	Cradle to gate	Electricity, fuel, farm operation, transportation, fertilizer and pesticide production and use, agricultural machinery

Reference	Article type	Language	Food assessed	System boundary	Sources of emissions
Hokazono S, Hayashi K. Variability in environmental impacts during conversion from conventional to organic farming: A comparison among three rice production systems in Japan. J Clean Prod. 2012;28:101–12.	Journal article	English	Brown rice	cradle to gate	Fertilizer, compost, pesticide, fuel use, machinery, rice-duck, seed, emission from paddy fields
Hokazono S, Hayashi. K, Sato M. Potentialities of organic and sustainable rice production in Japan from a life cycle perspective. Agron Res. 2009; 7:257–262.	Journal article	English	Brown rice	Cradle to gate	Soil conditioner, chemical fertilizer, herbicide, drying manufacturing of machines, chemicals, other materials, methane emissions from paddy fields
Honma T, Masuyama F. Greenhouse gas emission from paddy-rice production in Saitama Prefecture. Bulletin of the Saitama Prefectural Agriculture and Forestry Research Center. 2011.	Journal article	Japanese	Brown rice	Cradle to gate	Machinery, agricultural materials, production and use of fertilizer and pesticide, seed production, fuel, electricity, emissions from paddy fields
Iida S, Kato H, Niimura M, LIU Y, Istubo N, Watanabe K. Carbon footprint of boiled fish paste sasa-kamaboko in Miyagi Prefecture. Miyagi Prefect Rep Fish Sci. 2012;7–11.	Report	Japanese	Boiled fish paste	Cradle to point of use	Production of ship and fishing gear, electricity, fuel for transportation and operation, seasoning production
Ikeda T, Yoshikawa N, Matsuno T, Hasebe M, Maeda K, Amano K, Shimada K. Carbon Footprint calculation considering various cultivation methods for Koshihikari produced in Shiga prefecture. 6th Meet Inst Life Cycle Assessment, Japan. 2010;2010:134.	Conference proceeding	Japanese	White rice	Cradle to gate	Electricity, fuel, seeds, production and use of fertilizer and pesticide, packaging materials, emissions from paddy fields

Reference	Article type	Language	Food assessed	System boundary	Sources of emissions
Ikeda T, Yoshikawa N, Matsuo T, Hasebe M, Maeda K, Amano K, Fumoto T. Comprehensive assessment on environmental and economical impact of ecologically cultivated rice. The 7th meeting of the institute of Life Cycle Assessment, Japan. 2012. p. 160–1.	Conference proceeding	Japanese	White rice	Cradle to gate	Electricity, fuel, seeds, production and use of fertilizer and pesticide, packaging materials, emissions from paddy fields
Izumi A, Nakamura N, Hayashi K, et al. LCA of edamame considering the mitigation effects of packaging on food quality deterioration. In: *The 13rd Meet Inst Life Cycle Assessment, Japan.*; 2018:126-127.	Conference proceeding	Japanese	Edamame	Cradle to grave	(Background data from IDEA v2 was used. Detailed about emission source was not mentioned in the manuscript.)
Kawashima Y, Yoshikawa N. Report on Carbon Footprint of Rice (Variety : Koshihikari made in Shiga Prefecture). J Life Cycle Assessment, Japan. 2010;6:229–33.	Journal article	Japanese	White rice	Cradle to grave	Seeding production, f production and use of fertilizer and pesticide, fuel, electricity, packaging material, emission from paddy field
Koga N, Sawamoto T, Tsuruta H. Life cycle inventory-based analysis of greenhouse gas emissions from arable land farming systems in Hokkaido, northern Japan. Soil Sci plant Nutr. Melbourne. 2006;52:564–74.	Journal article	English	Cabbage	Cradle to gate	Liming, production and use of fertilizer, herbicide, and pesticide, transportation, farm operation, emission from soil, fuel, electricity
Liang R, Taniguchi K, Kawashima H, Kikuchi E, Soma T. Estimation of global warming emissions associated with a pig production system by life cycle assessment. J Life Cycle Assessment, Japan. 2007;3:178–83.	Journal article	Japanese	Pork	Cradle to gate	Feeding production, feeding transportation, farm operation (fuel use, electricity)

Reference	Article type	Language	Food assessed	System boundary	Sources of emissions
Maruyama K, Gocho N, Moriya T, Hayashi K. Life cycle assessment of super high-yield and conventional rice production systems. J Life Cycle Assessment, Japan. 2009;5:432–8.	Journal article	Japanese	Brown rice	Cradle to gate	Machinery, fuel, electricity, seed production, production and use of fertilizer and pesticide, emission from paddy fields
Masuda K, Takahashi Y, Yamamoto Y, Demura K. Life cycle assessment of low-input dairy farming: the case of "my-pace dairy farming" in the Korean region in Hokkaido. J Japanese Agric Syst Soc. 2005;21:99–112.	Journal article	Japanese	Milk	Cradle to gate	Production and transportation of feed, electricity, fuel, manure management, enteric fermentation
Masuda K, Tomioka M. Evaluation of greenhouse gas emission from tea cultivation systems using the life cycle assessment method. Japanese J Farm Manag. 2011;49:97–102.	Journal article	Japanese	Tea leaves	Cradle to gate	Electricity, fuel, production and use of fertilizer and pesticide, agricultural materials, farm operation
Masuda K, Tomioka M. Life cycle assessment of greenhouse gas emissions from environmentally friendly rice production. J Rural Probl. 2013;49:2219–24.	Journal article	Japanese	Brown rice	Cradle to gate	Electricity, fuel, production and use of fertilizer and pesticide, agricultural operation, emission from paddy fields
Masuda K. Measuring eco-efficiency of wheat production in Japan: a combined application of life cycle assessment and data envelopment analysis. J Clean Prod. 2016;126:373–81.	Journal article	English	Wheat	Cradle to gate	Seed, pesticide, fertilizer, fuel, electricity, agricultural service, agricultural machinery, land improvement, buildings, emission from paddy fields

Reference	Article type	Language	Food assessed	System boundary	Sources of emissions
Masuda K. Optimization Model for Mitigating Global Warming at the Farm Scale: An Application to Japanese Rice Farms. Sustainability. 2016;8.	Journal article	English	Brown rice, wheat, and soybeans	Cradle to gate	Seed production, pesticide, fertilizer, fuel, electricity, agricultural service, shipping bag, steel-framed building, timber-framed building. steel pipe greenhouse, agricultural machinery, plastic material, Land improvement and water use, emission from paddy fields, drying and storage facilities
Matsuura E, Komatsuzaki M, Hashimi R. Assessment of Soil Organic Carbon Storage in Vegetable Farms Using Different Farming Practices in the Kanto Region of Japan. Sustainability. 2018;10.	Journal article	English	Egg plant	Cradle to gate	Energy use, fertilizers, agrochemicals, and plastic materials during the vegetable cultivation period
Miki A, Nakatani J, Hirao M. Scenario analysis of drinking water usage applying life-cycle assessment for consumers. Environ Sci. 2010;23:447–58.	Journal article	Japanese	Water	Cradle to grave	Fuel use during packaging production, sale, distribution, water sampling
Mithraratne N, Barber A, Mclaren SJ. Carbon Footprinting for the Kiwifruit Supply Chain – Report on Methodology and Scoping Study Final Report. Wellington, New Zealand; 2010.	Report	English	Kiwifruit	Cradle to gate	Fuel use for mowing、Spraying, shelter trimming and mulching, fertiliser, electric use,
Nakamura T, Goto N. Life cycle assessment of beef cattle breeding in grazing system. 5th Meet Inst Life Cycle Assessment, Japan. 2009;179.	Conference proceeding	Japanese	Beef	Cradle to gate	Electricity, water, fuel, feed production and transportation, manure management

Reference	Article type	Language	Food assessed	System boundary	Sources of emissions
Ogino A, Ishida M, Ishikawa T, Ikeguchi A, Waki M, Yokoyama H, Tanaka Y, Hirooka H. Environmental impacts of a Japanese dairy farming system using whole-crop rice silage as evaluated by life cycle assessment. Anim Sci J. 2008;79:727–36.	Journal article	English	Milk	Cradle to gate	production and combustion of fossil fuels and feed transport, electricity
Ogino A, Oriito H, Shimada K, Hirooka H. Evaluating environmental impacts of the Japanese beef cow-calf system by the life cycle assessment method. Anim Sci J. 2007;78:424–32.	Journal article	English	Beef	Cradle to gate	Production and combustion of fossil fuels and feed transport, feed, electricity
Ogino A, Osada T, Takada R, Takagi T, Tsujimoto S, Tonoue T, Matsui D, Katsumata M, Yamashita T, Tanaka Y. Life cycle assessment of Japanese pig farming using low-protein diet supplemented with amino acids(Mitigation of methane and nitrous oxide emissions from livestock waste management). Soil Sci Plant Nutr. 2013;59:107–18.	Journal article	English	Pork	Cradle to gate	Feed production (including emission from field, chemical and organic fertilizer), fuel, electricity, feed production and transportation
Ohara S, Fukushima Y, Sugimoto A, Terajima Y, Ishida T, Sakoda A. Reduction in greenhouse gas emissions from process retrofitting and cultivar improvement in combined sugar-ethanol production from sugarcane. J Life Cycle Assessment, Japan. 2009;5:439–45.	Journal article	Japanese	Sugar	Cradle to factory gate	Fertilizer and pesticide use, fuel, electricity
Opio C, Gerber P, Mottet A, Falcucci A, Tempio G, MacLeod M, Vellinga T, Henderson B, Steinfeld H. Greenhouse gas emissions from ruminant supply chains–A global life cycle assessment. Food and agriculture organization of the United Nations. Rome, Italy; 2013. 1-214 p.	Report	English	Beef, and milk	Cradle to retail	Feed production and transportation, transport of animals and product (milk) processing into primary products, refrigeration, production of packaging material

Reference	Article type	Language	Food assessed	System boundary	Sources of emissions
Seo Y, Someya Y, Dowaki K. Environmental impacts and consumer preference for sustainably cultivated Japanese mustard spinach, komatsuna. J Environ Manage. 2019: 231:364–369.	Journal article	English	Japanese mustard spinach (*komatsuna*)	Cradle to gate	Dried manure, fuel, fertilizers, pesticides,
Shimura M, Takahashi H, Ito C, Shibuya M, Hayashi K, Matsumori K. Improvement potential of life cycle greenhouse gas emissions from paddy fields: assessing influence of sparse transplanting and non-puddling. Japan Agric Res Q. 2017;51:155–64.	Journal article	English	Rice	Cradle to gate	Fuel, agricultural machinery, electricity, steel greenhouse, fertilizer, pesticide, seeding, nursery box, bed soil, grain sack, water
Tsuiki M, Saitoh K, Maeda T. Life Cycle Assessment of Yearly Changes in Environmental Impacts of Japanese Dairy Farming. J Japanese Agric Syst Soc. 2009;25:185–94.	Journal article	Japanese	Milk	Cradle to gate	Fertilizers, pesticides, materials, machinery, facilities, fossil fuels, enteric fertilization, manure management
Tsutsumi M, Nakamura Y-N, Kaneko M, Hayashi Y, Tsubomi H, Yamada A, Kobayashi R. Life cycle impact assessment of the year-round grazing system for fattening Japanese brown beef steers. J Warm Reg Soc Anim Sci Japan. 2017;60:27–35.	Journal article	Japanese	Beef	Cradle to gate	Feed production, processing, and transportation, animal management, manure management, enteric fertilization
Tsutsumi M, Ono Y, Ogasawara H, Hojito M. Life-cycle impact assessment of organic and non-organic grass-fed beef production in Japan. J Clean Prod. 2018;172:2513–20.	Journal article	English	Beef	Cradle to gate	Feed production, processing, and transportation, animal management, enteric fermentation, and manure management
Tyedmers P, Parker R. Fuel Consumption and Greenhouse Gas Emissions from Global Tuna Fisheries : A preliminary assessment. ISSF Tech Rep. 2012.	Grey report	English	Tuna	Cradle to gate	Fuel use during fishing, transport, vessel construction, gear and bait provision, refrigeration

Reference	Article type	Language	Food assessed	System boundary	Sources of emissions
Yamamoto Y, Nakayama T, Watanabe M, Itsubo N. Carbonfootprint and Waterfootprint for coffee and tea. 6th Meet Inst Life Cycle Assessment, Japan. 2011;272-273.	Conference Proceeding	Japanese	Coffee	Cradle to gate	Coffee cultivation and transportation, packaging production, waste, cooking. management
Yoshikawa N, Amano K, Shimada K. Evaluation of environmental load on fruits and vegetables consumption and its reduction potential. Environ Syst Res. 2007;35:499–509.	Journal article	Japanese	Potato, satoimo, cabbage, spinach, leek (*negi*), Chinese cabbage (*hakusai)*, lettuce, daikon, carrot, tomato, pepper, eggplant, cucumber, orange (*unsyu*), Japanese pear, grape, persimmon (*kaki*), water melon, melon, peach, and strawberry	Cradle to grave	Fetirizers, Emission from paddy field, facilities use, transportation, fuel, energy use at retail, gas and water use at home
Yoshikawa N, Ikeda T, Amano K, Fumoto T. Life-cycle assessment of ecologically cultivated rice applying DNDC-Rice model. Colledge Sci Eng Ritsumeikan Univ. 2012;598:7–9.	University Bulletin	English	Rice	Cradle to gate	Emission from paddy field, fertilizer, manure, fuel, electricity, transportation, waste treatment
Yoshikawa N, Ikeda T, Amano K, Shimada K. Carbon footprint estimation and data sampling method: a case study of ecologically cultivated rice produced in Japan. VII Intenational Conference of Life Cycle Assessment in Agro-food sectors. Bari, Italy; 2010.	Conference proceeding	English	Rice	Cradle to grave	Energy, fertilizer, agrochemicals, packaging materials, seeds, emissions from paddy field, transportation, water supply

Reference	Article type	Language	Food assessed	System boundary	Sources of emissions
Yue Y, Narita N, Sugai M, Ogawa K. Carbon Footprint of noodles. 7th Meet Inst Life Cycle Assessment, Japan. 2012;143-144.	Conference proceeding	Japanese	Udon noodle	Cradle to grave	Production of ingredients, electricity
Ziegler F, Winther U, Hognes ES, Emanuelsson A, Sund V, Ellingsen H. The Carbon Footprint of Norwegian Seafood Products on the Global Seafood Market. J Ind Ecol. 2013;17:103–16.	Journal article	English	Salmon, and mackerel	Cradle to wholesaler	Diesel consumption, refrigerants emission in fishing, production of agricultural and marine ingredients, deed production, processing, transport packaging

Supplemental Table 2. Number of article assessed the greenhouse gas emission of each type of food

Food category	Number of article	Food name (number of article)
Cereal	18	Rice* (18), wheat (3), and udon noodle (1),
Potato	1	Potato (2) and satoimo (1)
Sugar	1	Sugar (1)
Beans	1	Soybean (1)
Seeds	0	-
Vegetables	5	Cabbage (2), spinach (1), leek (*negi*) (1), Chinese cabbage (*hakusai*) (1), lettuce (1), daikon (1), carrot (1), tomato (1), pepper (1), eggplant (2), cucumber (1), Japanese mustard spinach (*komatsuna*) (1), and edamame (1)
Fruits	1	Orange (*unsyu*)(1), Japanese pear (1), grape (1), persimmon (*kaki*) (1), watermelon (1), melon(1), peach (1), and strawberry (1)
Mushrooms	0	-
Seaweed	0	-
Fish and seafood products	3	Boiled fish paste (1), tuna (1), salmon (1), and mackerel (1)
Meat	8	Beef (6), and pork (4)
Eggs	0	-
Dairy	4	Milk (3)
Fat and oils	0	-
Confectionary	0	-
Beverage	5	Coffee (2), water (1), and citrus juice (1)

*Both write rice and brown rice were included.

Supplemental Table 3. Greenhouse gas emission (GHGE) value for post-farm stage

Stage	GHGE value	unit	References
Rice			
Rice polishing	0.05	kg CO_2-eq/kg	[1]
Rice distribution and retailing	0.31	kg CO_2-eq/kg	[1]
Rice cooking	0.31	kg CO_2-eq/kg	[1]
Rice waste treament	0.01	kg CO_2-eq/kg	[1]
Meat and fish products			
Animal slaughtering (beef)	0.17	kg CO_2 eq/kg-meat	[2, 3]
Animal slaughtering (pork)	0.12	kg CO_2 eq/kg-meat	[2]
Animal slaughtering (chicken)	0.04	kg CO_2 eq/kg-meat	[2]
Meat packaging	0.6	kg CO_2 eq/kg-meat	[2]
Meat Processing	0.66	kg CO_2-eq/kg	[2]
Transportation, distribution and shopping (beef)	0.23	kg CO_2-eq/kg	[2]
Transportation, distribution and shopping (pork)	0.24	kg CO_2-eq/kg	[2]
Transportation, distribution and shopping (chicken)	0.24	kg CO_2-eq/kg	[2]
Aging, storage and display (beef)	0.06	kg CO_2-eq/kg	[2]
Aging, storage and display (pork)	0.07	kg CO_2-eq/kg	[2]
Aging, storage and display (chicken)	0.07	kg CO_2-eq/kg	[2]
Post farm (transport, processing and refrigeration of products) (milk and dairy)	0.17	kg CO_2-eq/kg	[3]*
Fish processing	1.25	kg CO_2-eq/kg fillet	[4, 5]
Other foods			
Processing vegetables	0.07	kg CO_2-eq/kg	[6]
Packaging	0.06	kg CO_2-eq/kg	[6]
Transport to regional distribution center	0.13	kg CO_2-eq/kg	[6]
Retail	0.1	kg CO_2-eq/kg	[6]

GHGE, greenhouse gas emission.

*Six products were considered (processed milk, cheese, whey, yoghurt, skimmed milk powder and whole milk powder)

[1] Yoshikawa N, et al. VII Intenational Conference of Life Cycle Assessment in Agro-food sectors. Bari, Italy; 2010.

[2] Roy P, et al. J Environ Manage. 2012;93:218–24.

[3] Opio C, et al. Greenhouse gas emissions from ruminant supply chains–A global life cycle assessment. FAO, Rome, Italy; 2013

[4] Burg v.d. S.W.K. van den, Taal C., Boer de, I.J.M., Bakker T., Viets T.C. (2012)

[5] Environmental performance of wild-caught North Sea whitefish, a comparison with aquaculture and animal husbandry using LCA. LEI, Den Haag. Report no. 2011-090

[6] Clune S, et al. J Clean Prod. 2017;140:766–83.

Supplemental Table 4. The national database used for unit price complement of input-output table

Food group	Database	Static	Publisher
Cereals	Crop survey*, rice and wheat (2005)	yeild (t)	MAFF
Potatoes	Crop survey*, potatoes (2005)<br>Crop survey*, vegetables (2005)	yeild (t)	MAFF
Beans	Crop survey*, beans (2005)	yeild (t)	MAFF
Vegetables (exc. potatoes)	Crop survey*, vegetables (2005)<br>Food supply and demand table† (2005)	yeild (t)	MAFF
Fruits	Crop survey*, fruit (2005)	yeild (t)	MAFF
Mashrooms	Forestry products survey‡ (2005)	yeild (t)	MAFF
Meat	Livestock census§ (2005)		MAFF
Egg	Livestock census, Egg distribution survey§ (2005)	production (t)	MAFF
Seafood	Fishery production survey|| (2005)	production value (JPY) and volume (t)	MAFF
Alcoholic beverages and salt	Census of Manufactures¶	shipment value and quantity (t or kl)	MIC

MAFF, Ministry of Agriculture, Forestry and Fisheries; MIC, Ministry of Internal Affairs and Communications

*"*Sakkyo chosa*" in Japanese. Data was provided by Ministry of Agriculture, Forestry and Fisheries (http://www.maff.go.jp/j/tokei/kouhyou/sakumotu/index.html, accessed March 14, 2019)

†"*Shokuryo jukyu hyo*" in Japanese. Data was provided by Ministry of Agriculture, Forestry and Fisheries (http://www.maff.go.jp/j/zyukyu/fbs/, accessed March 14, 2019)

‡"Tokuyo Rinsanbutsu Seisan Toukei Chosa" in Japanese. Data was provided by Ministry of Agriculture, Forestry and Fisheries (http://www.maff.go.jp/j/tokei/kouhyou/tokuyo_rinsan/, accessed March 14, 2019)

§"*Tikusan Tokei Chosa*" in Japanese. Data was provided by Ministry of Agriculture, Forestry and Fisheries (http://www.maff.go.jp/j/tokei/kouhyou/tikusan/, accessed March 14, 2019)

|| "Kaimengyo-gyo Seisan Toukei Chosa" in Japanese. Data was provided by Ministry of Agriculture, Forestry and Fisheries (http://www.maff.go.jp/j/tokei/kouhyou/kaimen_gyosei/, accessed March 14, 2019)

¶"*Kougyo-Toukei*" in Japanese. Data was provided by Ministry of Internal Affairs and Communications (http://www.meti.go.jp/statistics/tyo/kougyo/index.html, accessed March 14, 2019)

References

1. Science and Technology Agency (2015) *Standard Tables of Food Composition in Japan. (in Japanese)*. 7th editio. Printed Bureau of Ministry of Finance.
2. Nansai K, Kagawa S, Kondo Y, et al. (2009) Improving the completeness of product carbon foodprints using a global link input-output model: The case of Japan. *Econ. Syst. Res.* **9**, 267–290.
3. IPCC (2014) *Climate Change 2014: Synthesis Report. Contribution of Working Groups I, II and III to the Fifth Assessment Report of the Intergovernmental Panel on Climate Change*. Geneva, Switzerland.
4. Barilla Center for Food & Nutrition (2011) *Double Pyramid: healthy food for people, sustainable food for the planet*.
5. Barilla Center for Food & Nutrition (2016) *Double pyramid 2016 a more sustainable future depends on us*.
6. Yoshikawa N, Ikeda T, Amano K, et al. (2010) Carbon footprint estimation and data sampling method: a case study of ecologically cultivated rice produced in Japan. *VII Intenational Conference of Life Cycle Assessment in Agro-food sectors*
7. Nansai K, Kondo Y, Kagawa S, et al. (2012) Estimates of embodied global energy and air-emission intensities of japanese products for building a Japanese input-output life cycle assessment database with a global system boundary. *Environ. Sci. Technol.* **46**, 9146–9154.
8. Charrondiere UR, Haytowit D & Stadlmayr B (2012) Density Database Version 2 . 0. *Fao/Infoods* **2**, 24.
